# Spatial learning by mice in three dimensions

**DOI:** 10.1016/j.bbr.2015.04.035

**Published:** 2015-08-01

**Authors:** Jonathan J. Wilson, Elizabeth Harding, Mathilde Fortier, Benjamin James, Megan Donnett, Alasdair Kerslake, Alice O’Leary, Ningyu Zhang, Kate Jeffery

**Affiliations:** Institute of Behavioural Neuroscience, Research Department of Experimental Psychology, Division of Psychology and Language Sciences, University College London, United Kingdom

**Keywords:** Navigation, 3D, Mice, Memory

## Abstract

We tested whether mice can represent locations distributed throughout three-dimensional space, by developing a novel three-dimensional radial arm maze. The three-dimensional radial maze, or “radiolarian” maze, consists of a central spherical core from which arms project in all directions. Mice learn to retrieve food from the ends of the arms without omitting any arms or re-visiting depleted ones. We show here that mice can learn both a standard working memory task, in which all arms are initially baited, and also a reference memory version in which only a subset are ever baited. Comparison with a two-dimensional analogue of the radiolarian maze, the hexagon maze, revealed equally good working-memory performance in both mazes if all the arms were initially baited, but reduced working and reference memory in the partially baited radiolarian maze. This suggests intact three-dimensional spatial representation in mice over short timescales but impairment of the formation and/or use of long-term spatial memory of the maze. We discuss potential mechanisms for how mice solve the three-dimensional task, and reasons for the impairment relative to its two-dimensional counterpart, concluding with some speculations about how mammals may represent three-dimensional space.

## Highlights

• Mouse spatial memory was tested on a novel 3D radial arm maze.
• Mice exhibited learning on working and reference memory tasks on the 3D maze.
• Working memory was not impaired on the 3D maze when compared with a 2D analogue.
• Reference memory was impaired on the 3D maze when compared with the 2D maze.
• This may be explained by a differential encoding of vertical and horizontal space.

## 1 Introduction

The ability to accurately navigate through the world is vital for the survival of mobile animals, and requires the perception and encoding of spatial cues associated with important locations such as food nesting location. Studies over many years have concluded that mammals create an internal representation of space (sometimes known as the cognitive map; [1]). This work has primarily focused on spatial navigation in horizontal planar environments: however, recently, interest has been growing in the means by which larger and more dimensionally complex spaces might be represented and used in navigation (see Jeffery et al. [2] for review). Few laboratory studies of three-dimensional spatial encoding have been conducted to date, and so the aim of the present experiment was to determine whether mice could perform a 3D version of a widely used laboratory-based navigation task, the radial maze task [3].

The advantage of the Olton radial maze paradigm is that it can test both long-term (reference) and short-term (working) memory concurrently. In the 3D version of the maze, which because of its spherical symmetry we have named the radiolarian maze,^1^ food rewards are located at the end of arms that project from a sphere, rather than the usual disc, so that for each horizontal arm coordinate there are two vertical coordinates. Thus, animals have to remember the distribution of rewards by encoding both horizontal and vertical components simultaneously, and also dynamically update this representation as trials progressed and the arms become depleted. We used mice because they are lightweight, are skilled climbers and have a naturally 3D ecology.

The animals were trained on two versions of the radial maze task, the standard version in which all arms begin by being baited and are depleted without replacement as the trial progresses, and a reference memory task in which only some of the arms are ever baited. We reasoned that if mice cannot encode the vertical component of the space then they should have difficulty learning both versions of the task due to confusion between the upper and lower arms at a given azimuth (horizontal direction). Comparisons with a version of the original radial arm maze and with a two-dimensional analogue of the radiolarian maze revealed that mice are equally able to learn the working memory version of the task in the radiolarian maze as in two-dimensional mazes. In the reference memory task the rate of reduction in reference memory errors were comparable for the radiolarian maze and its two-dimensional analogue; however, overall learning was reduced in the radiolarian maze. We suggest that mice are able to simultaneously represent both vertical and horizontal components of a spatial task, but that having to encode both components creates difficulties for them. We conclude with a discussion of how the 3D structure of the maze task may be represented.

## 2 Methods

### 2.1 Subjects

Subjects were 40 male C57BL/6J mice obtained at 8–10 weeks of age from Charles River Laboratories, individually housed and mildly food restricted to maintain body weight at 90% of free feeding weight. A 12 h reversed light/dark cycle was used with 30 min simulated dawn at 23:30 and simulated dusk at 11:30; all mice were trained during their dark cycle between 12:30 and 15:00. Experiment 1 used three cohorts of mice (each *n* = 8) while Experiment 2 used two (each *n* = 8). All mice were naïve to the experimental apparatus prior to habituation. All procedures carried out during these experiments were licensed by the UK Home Office, subject to the restrictions and provisions contained in the Animals (Scientific Procedures) Act of 1986.

### 2.2 Apparatus

**Fig. 1:**
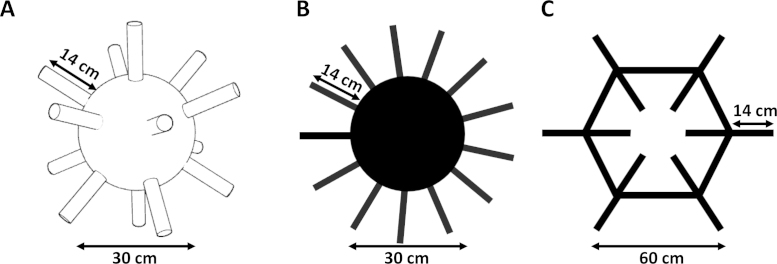
(A) Radiolarian maze; (B) classic maze; (C) hexagon maze.

The apparatus comprised three versions of the classic radial arm maze: a 3D version named the radiolarian maze (Fig. 1A), a 13-arm version referred to throughout as the classic maze (Fig. 1B), and a two-dimensional analogue of the radiolarian maze, named the hexagon maze (Fig. 1C). Arms were baited with condensed milk applied to dressmakers’ pins inserted at the end of each arm. All three mazes were used for Experiment 1, and just the radiolarian and hexagon mazes for Experiment 2. All experiments were carried out in the same well lit room, with consistent visual extramaze cues available to mice throughout data collection.

The radiolarian maze (Fig. 1A) was constructed from lightweight materials and coated with papier-mâché followed by crèpe bandage, to provide grip. The central section comprised a 30 cm diameter sphere from which radiated 14 equidistantly placed cylindrical arms, each 14 cm in length and 3.5 cm in diameter. The maze was suspended by nylon line in the centre of an empty 19-inch rack, with the lowermost arm 30 cm above the floor of the rack.

The classic maze (Fig. 1B) was a 13-arm version of the standard Olton radial arm maze, constructed using MDF and covered with crèpe bandage so as to maintain consistency with the radiolarian maze. The central section comprised a 30 cm diameter circle from which radiated 13 evenly spaced arms, each 14 cm in length and 3.5 cm in diameter. The maze was raised 30 cm above a table, and was placed in the centre of the experimental room.

The hexagon maze (Fig. 1C), a two-dimensional analogue of the radiolarian maze, had 12 arms. The maze constituted a hexagonal ring, with 30 cm sides each 3.5 cm in width, with six 14 cm arms, with a width of 3.5 cm, extended outwards and six 14 cm arms extended inwards from the corners of the ring. Thus, on returning from an arm excursion mice would have four choices—turn left, turn right, go straight ahead or turn back. This maze was therefore more geometrically similar to the radiolarian maze than was the classic maze. The maze was covered in crèpe bandage, so as to maintain consistency with the radiolarian and classic mazes, and was again raised 30 cm above a table in the centre of the experimental room.

### 2.3 Habituation

For both experiments, subjects were habituated to each maze for 5 days before training commenced. In the first 2 days no arms were baited and mice were allowed to freely traverse the maze for 15 min. In the final 3 days each mouse was introduced to the maze from each of the arms in turn. Once it made its way from an arm to the centre of the maze it was removed and placed on another randomly selected arm. This was repeated until all animals willingly navigated from each of the arms to the centre within 1 min.

### 2.4 Experiment 1—working memory task

#### 2.4.1 Subjects and training

Each cohort of mice (*n* = 8 per cohort) was trained on one of the three mazes only.

Once habituation was completed, the working memory phase of training began. For the working memory task, all of the arms of each of the mazes were baited with condensed milk. Mice were required to retrieve food from all arms of the maze. An arm visit was only recorded when an animal's head reached the end of an arm. The number of re-entry errors (repeated visits to an already-depleted arm) and omission errors (number of unvisited arms) and order of visits were scored manually by two experimenters, who sat in opposing corners of the experimental room. Mice were removed from the maze after either 15 min or once they had collected the food reward from all arms of the maze, whichever was soonest. Mice were trained on this task for one trial per day for at least 7 days or until the number of omission and re-entry errors had reached a 3-day plateau, defined as a non-significant difference between the final 3 days of trials (using repeated measures ANOVA).

#### 2.4.2 Analysis

Paired *t*-tests comparing the first three trials to the last three trials were used to assess learning. Values for task latency, the total number of visits, the number of omission errors and the rate of re-entry (working memory) errors as a percentage of total visits were compared between mazes using repeated measures ANOVA.

For the movement pattern analysis on the radiolarian maze, the lower vertically projecting arm was removed from analysis as this arm was only ever visited by two mice, and was consistently the last to be visited. For comparison of movements within and between layers, seven arms comprised the upper layer—the upwards projecting arm at the top of the maze and the six outwards projecting arms on the top section of the maze. The six arms projecting outwards in the lower half of the maze comprised the lower layer. The percentages of total visits that could be assigned to within- and between-layer movements were then calculated as a proportion of chance for each movement type, with a chance level of 50% for both within-layer and between-layer movements, as there was an overall 6.5/13 chance of movement between layers (due to the seven arms present on the upper layer, and six arms on the lower layer), and a 6.5/13 chance of movement within layers.

Further analyses of movement patterns tested for neighbouring-arm biases. In the radiolarian maze, on the upper layer the animals could visit one of seven neighbouring arms (two horizontally adjacent, two diagonally adjacent, one directly below the current arm, one vertically oriented arm, or an immediate revisit to the just-visited arm). When on the lower layer animals could visit one of six neighbouring arms (two horizontally adjacent, two diagonally adjacent, one directly above the current arm, or an immediate revisit).

In the classic maze, three out of 13 arms were neighbouring: the two immediately adjacent arms, and the just-visited arm.

In the hexagon maze, six out of 12 arms were neighbouring: the arm opposite the previously visited arm, two diagonally adjacent arms, two adjacent arms which were within the same subset of arms (i.e. moving from an outwards projecting arm to one of the two adjacent outwards projecting arms), and the just-visited arm. Arm visits were expressed as a proportion of chance.

### 2.5 Experiment 2—reference memory task

#### 2.5.1 Subjects and training

Each cohort of mice (*n* = 8 per cohort) was trained on one of the two mazes only. Six baited arms were assigned to each mouse. In the radiolarian maze, only arms on the two central layers of the maze were baited (three upper and three lower). In the hexagon maze, three of the outer six arms and three of the inner six arms were baited. The six goal locations in each maze were specified in allocentric coordinates, such that the baited arms for each mouse were defined by their relation to extramaze cues. Each maze was rotated horizontally by 180° either every trial or every other trial to control for intramaze cues, such as tactile cues and olfactory cues related to scent marks left by the mice. For each maze, two trials were carried out per day over 25 days, with the first session commencing at 1 pm and the second at 3 pm. Each trial lasted for 5 min or until all six rewards had been retrieved, whichever was sooner.

Performance was scored as working memory errors (visits to already-visited arms) and reference memory errors (visits to never-baited arms). The order of arm visits, number of total visits, number of omission errors (baited arms that were not visited), task latency, and the total number of erroneous visits (commission errors) were also collected.

#### 2.5.2 Probe trials

Two 5-min probe trials were carried out the day after completion of the reference memory task, in order to rule out the (remote) possibility that the animals had used olfaction to find the food. All arms were left unbaited, and the maze was rotated 180° between probe trials to control for intramaze cues. Reference memory errors, re-entry errors, commission errors, omission errors, task latency, order of arm visits and the total number of visits were scored.

#### 2.5.3 Analysis

Values for task latency, the total number of visits, number of omission errors, and the percentages of reference memory, working memory and commission errors were analysed. Paired *t*-tests comparing the first three and last 3 days of training were used to assess learning. A between-subjects analysis compared the rate of learning for each of these variables between mazes.

## 3 Results

### 3.1 Experiment 1—working memory task

#### 3.1.1 Radiolarian maze

To assess learning, we compared the first and last 3 days of training. The four variables analysed were the total number of visits, task latency, omission errors, and re-entry errors, measured as a percentage of the total. Paired *t*-tests found no difference in the total number of arm visits between the first three trials (16 ± 2 visits) and final three trials (17 ± 1, *t*_(7)_ = −0.520, *p* = 619), but task latency decreased significantly from 842 ± 40 to 496 ± 68 s (*t*_(7)_ = 5.129, *p* = 0.001).

**Fig. 2:**
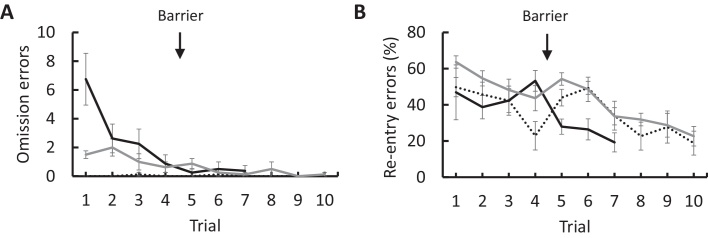
Comparison of working memory task learning between the radiolarian maze (black), classic maze (black dotted) and hexagon maze (grey). (A) Number of omission errors and (B) rate of arm-re-entry errors as a percentage of the total number of visits. Arrows represent introduction of barriers to the classic maze.

Omission errors (failures to enter one of the arms) decreased significantly from an average of 3.9 ± 1.2 errors on the first three trials to 0.4 ± 0.4 errors on the final three trials (*t*_(7)_ = 3.816, *p* = 0.007; Fig. 2A). Re-entry errors declined significantly from an average of 44.7 ± 7.9 to 24.5 ± 2.9% (*t*_(7)_ = 2.538, *p* = 0.039; Fig. 2B).

We finally analysed movement patterns, in light of findings from previous studies of a bias towards horizontal movements in three-dimensional environments [4,5]. We calculated the percentage of the total number of visits that were horizontal within-layer movements, and expressed these as a proportion of chance (chance = 50%). Within-layer movements did not exceed chance (proportion of chance: 1.04 ± 0.04, *t*_(7)_ = 0.756, *p* = 0.474), but there was a significant preference for visiting neighbouring arms regardless of layer (mean 1.4 ± 0.05, *t*_(7)_ = 8.20, *p* < 0.001).

In conclusion, mice showed good working memory performance on the radiolarian maze, showing a decrease in task latency, omission errors and re-entry errors. Thus, they were able to represent the spatial aspects of the task and track these across trials, even though the arms were distributed in 3D space, and each horizontal arm position occurred at two vertical locations; they did not appear to use stereotyped choice strategies and did not show differences between horizontal and vertical movement patterns.

#### 3.1.2 Classic maze

Because of the unexpectedly good performance of the animals on the 3D maze, we conducted a classic-maze experiment, using a 13-arm version of a standard radial maze, in order to develop a benchmark of standard two-dimensional performance against which to compare the radiolarian maze findings. Mice received 10 days of trials.

Observation of the animals during learning revealed a marked propensity for the mice to adopt stereotyped movement patterns, showing a strong preference for visiting neighbouring arms, unlike the patterns typically shown by rats in a similar apparatus [6]. Over the first four trials the proportion of visits to neighbouring arms increased from 0.9 ± 0.1 on the first trial to 3.0 ± 0.3 on the fourth trial. One sample *t*-tests for all 10 trials combined revealed a significantly above-chance preference for visiting neighbouring arms (mean 2.3 ± 0.18, *t*_(7)_ = 7.28, *p* < 0.001).

To thwart use of an adjacent-arm rule, we introduced barriers to the entrance of each arm on the fifth day to try and reduce stereotyped behaviour. While this initially reduced the proportion of neighbouring arm visits to 1.4 ± 0.3 on the fifth trial, animals quickly returned to this stereotyped behaviour, reaching a peak proportion of 3.4 ± 0.3 neighbouring arm visits by trial 10.

There was no difference in the number of total visits between the first three trials (27 ± 2) and the final three trials (19 ± 2, *t*_(7)_ = 2.254, *p* = 0.059). There was, however, a decrease in task latency from the first three trials (412 ± 49 s) to the final three trials (210 ± 49 s, *t*_(7)_ = 3.505, *p* = 0.010).

Owing to the stereotypy discussed above, mice were able to complete the working memory task with zero omission errors from the first trial, with an average of 0.04 ± 0.04 omission errors first three trials and an average of 0 ± 0 omission errors on the final three trials (Fig. 2A). These values were not significantly different (*t*_(7)_ = 1.000, *p* = 0.351). Re-entry errors between the first three trials (45.9 ± 4.9%) and the final three trials (23.1 ± 6.0%) almost approached significance (*t*_(7)_ = 2.350, *p* = 0.051; Fig. 2B).

In summary, mice completed the working memory task on the classic maze without omission errors and with no decrease in re-entry errors, while movement pattern analysis indicated a high tendency to choose neighbouring arms in a stereotyped manner, which accounted for the error pattern. To overcome this problem and assess true spatial learning, we adapted the maze so as to reduce the affordance of the maze for stereotypy and make it structurally more similar to the radiolarian maze, while retaining the two-dimensional layout. Results from this “hexagon maze” are described in the following section.

#### 3.1.3 Hexagon maze

The hexagon maze is more akin to the radiolarian maze in which the animals had multiple neighbouring arms to choose from and we hoped it would prevent, or at least reduce, the application of simple rules. Mice received 10 days of trials on this maze, and analysis proceeded as before.

There was a significant decrease in the total number of arm-visits (*t*_(7)_ = 5.667, *p* = 0.001), from 26 ± 2 visits on the first three trials to an average of 17 ± 1 on the final three trials. There was also a decrease in task latency from 776 ± 31 to 269 ± 91 s (*t*_(7)_ = 6.396, *p* < 0.001). Omission errors decreased from an average of 1.5 ± 0.4 to 0.2 ± 0.2 (*t*_(7)_ = 4.651, *p* = 0.002; Fig. 2A). Analysis of re-entry errors revealed that, unlike in the classic maze, there was evidence of spatial learning, with a significant decrease in the percentage of re-entry errors from an average of 55.5 ± 2.0 to 27.8 ± 3.9% (*t*_(7)_ = 6.905, *p* < 0.001; Fig. 2B). Nevertheless, movement pattern analysis found that despite the change in maze design, there remained a significant preference for visiting neighbouring arms (mean 1.5 ± 0.03, *t*_(7)_ = 17.13, *p* < 0.001).

#### 3.1.4 Comparison between mazes

We next carried out repeated measures ANOVAs to compare the learning between the three radial arm mazes. We focused on the two error types (omission errors and re-entry errors), followed by comparison of neighbouring-arm visits.

Comparison of omission errors between mazes for the first three versus last three trials revealed a significant reduction in the number of omission errors (*F*_(1,21)_ = 25.389, *p* < 0.001). Analysis of between-maze effects revealed a significant difference between mazes (*F*_(2,21)_ = 5.342, *p* = 0.013; Fig. 2A), in which Bonferroni adjusted post hoc comparisons revealed significantly fewer omission errors on the classic maze than the radiolarian maze (*p* = 0.012), with no differences between the hexagon and classic maze (*p* = 0.638) or hexagon and radiolarian mazes (*p* = 0.190). For re-entry errors, repeated measures ANOVA also showed a significant overall reduction (*F*_(1,21)_ = 28.801, *p* < 0.001) with no difference between mazes (*F*_(2,21)_ = 1.517, *p* = 0.242; Fig. 2B).

**Fig. 3:**
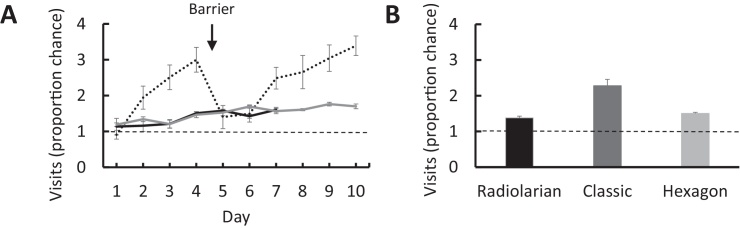
Movement patterns during the working memory task. (A) Neighbouring-arm visits on the radiolarian (black), classic (black dotted) and hexagon mazes (grey) for each day of trials, represented as a proportion of chance. Arrows represent the time points at which barriers were added to the classic maze. (B) Task average of neighbouring arm visits represented as a proportion of chance for each of the three mazes. Chance levels are represented by a horizontal dashed line.

Finally, comparisons of the neighbouring arm bias revealed a significant difference between mazes (*F*_(2,21)_ = 21.026, *p* < 0.001), with Bonferroni-corrected independent-groups *t*-tests (only *p* < 0.017 were considered to be significant) revealing a stronger preference for visiting neighbouring arms in the classic maze than the radiolarian (*t*_(7.97)_ = 4.951, *p* = 0.001) and hexagon mazes (*t*_(7.39)_ = 4.347, *p* = 0.003) and no difference between the radiolarian and hexagon mazes (*t*_(14)_ = 2.281, *p* = 0.039; Fig. 3A and B).

#### 3.1.5 Summary

Together, these data show that mice can hold a short-term working-memory representation of reward locations on the hexagon and radiolarian mazes, and that their ability to do so increases with experience of the task. Both the hexagon maze and radiolarian maze yielded similar results for reductions in working memory errors, and for neighbouring arm biases, suggesting that the greater geometric complexity of the mazes compared to the classic maze can reduce the behavioural biases observed on the latter.

We next looked at whether mice can form long-term spatial memory representations in three as well as two dimensions.

### 3.2 Experiment 2—reference memory experiment

This experiment compared long-term spatial memory, in addition to working memory, between the 3D radiolarian maze and its 2D counterpart, the hexagon maze. Sixteen naïve mice, split into two cohorts of *n* = 8 each, were trained on either the radiolarian maze or hexagon maze over 25 consecutive days, with two trials per day. Both cohorts of mice were able and motivated to move around their designated maze by the end of habituation. The classic maze was not used for the reference memory task, due to the strong neighbouring-arm bias observed in Experiment 1.

#### 3.2.1 Radiolarian maze

In the radiolarian maze, total arm visits did not decrease between the first 3 days (9 ± 1) and the last 3 days (10 ± 1, *t*_(7)_ = −0.768, *p* = 0.468) but there was a reduction in task latency from 295 ± 3  to 168 ± 22 s (*t*_(7)_ = 5.841, *p* = 0.001).

**Fig. 4:**
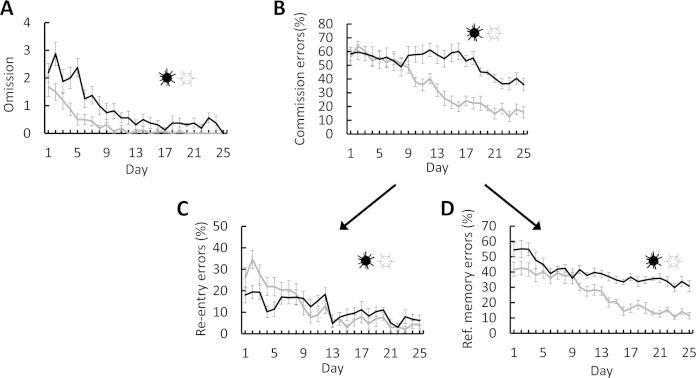
Comparison of learning rates between radiolarian maze (black) and hexagon maze (grey). (A) Omission errors; (B) commission errors; (C) working memory errors; (D) reference memory errors.

Omission errors decreased significantly from an average of 2.3 ± 0.3 errors on the first 3 days of trials to 0.3 ± 0.2 on the final 3 days of trials (*t*_(7)_ = 8.064, *p* < 0.001; Fig. 4A). Commission errors (percentage of all visits that were erroneous) declined significantly from 57.1 ± 2.8% on the first 3 days of trials to 37.7 ± 4.0% on the final 3 days of trials (*t*_(7)_ = 4.393, *p* = 0.003; Fig. 4B). These types of errors were further split into two component parts—re-entry errors and reference memory errors (visits to arms that had never been baited). The percentage of re-entry errors decreased significantly from 15.3 ± 2.5 to 7.0 ± 2.0% (*t*_(7)_ = 2.7, *p* = 0.032; Fig. 4C), while reference memory errors decreased from 56.0 ± 2.6 to 31.5 ± 2.9% (*t*_(7)_ = 7.141, *p* < 0.001; Fig. 4D). There was thus clear evidence of 3D spatial learning: working memory performance improved slightly and reference memory improved considerably across the course of training.

#### 3.2.2 Hexagon maze

Mice on the hexagon maze showed a significant reduction in the total number of visits between the first 3 days (13.0 ± 0.9) and the last 3 days (7.0 ± 0.3; *t*_(7)_ = 5.341, *p* = 0.001) and a significant reduction in task latency from 271 ± 14 to 104 ± 12 s (*t*_(7)_ = 14.878, *p* < 0.001).

Omission errors decreased from 1.5 ± 0.3 on the first 3 days to 0 ± 0 on the final 3 days (*t*_(7)_ = 5.029, *p* = 0.002; Fig. 4A). Commission errors decreased from 60.2 ± 3.0 to 15.52 ± 2.9% (*t*_(7)_ = 11.546, *p* < 0.001; Fig. 4B). Of these, the percentage of re-entry errors decreased from 29.4 ± 2.2 to 3.6 ± 1.7% (*t*_(7)_ = 8.943, *p* < 0.001; Fig. 4C), while the percentage of reference memory errors decreased from 41.9 ± 2.3 to 12.1 ± 1.7% (*t*_(7)_ = 5.44, *p* = 0.001; Fig. 4D).

#### 3.2.3 Probe trials

Probe trials were then conducted to rule out the use of olfactory cues in solving the task; it was expected that probe trial performance should differ from performance on the first day but not from performance on the last day, so these were examined with repeated-measures ANOVA comparing trial type (training/probe) against day (first/last). For the radiolarian maze, there was a main effect of trial type on the percentage of reference memory errors (*F*_(2,14)_ = 11.736, *p* = 0.001). Bonferroni-corrected paired *t*-tests (in which only values with a *p* < 0.017 were considered significant) found a significantly lower rate of reference memory errors on the final day when compared to the first day (*t*_(7)_ = 3.31, *p* = 0.013) and a significantly lower rate of reference memory errors on the probe day when compared to the first day (*t*_(7)_ = 4.03, *p* = 0.005). As expected, there was no difference between the final day of trials and the probe trials (*t*_(7)_ = 0.99, *p* = 0.354).

**Fig. 5:**
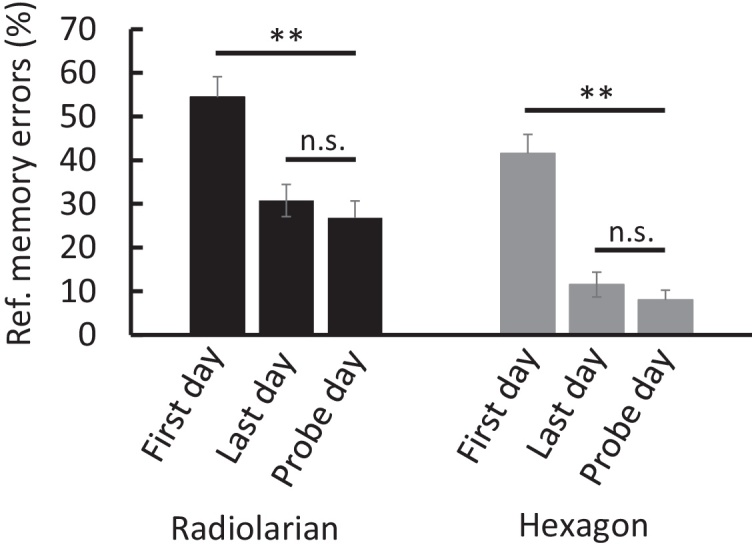
Probe trials to rule out reward detection (**p < 0.01; n.s. denotes non-significant).

For the hexagon maze, there was a main effect of trial type of the percentage of reference memory errors (*F*_(2,14)_ = 26.868, *p* < 0.001). Bonferroni-correct paired *t*-tests (*p* < 0.017 considered significant) showed a significantly lower rate of reference memory errors in the final day compared to the first day (*t*_(7)_ = 5.44, *p* = 0.001) and in the probe trials when compared to the first day (*t*_(7)_ = 5.34, *p* = 0.001), with again, no difference in probe trials compared to the final day of trials (*t*_(7)_ = 1.44, *p* = 0.194; Fig. 5).

Overall, then, probe trial performance was similar to performance by the end of training for both maze types.

#### 3.2.4 Comparison between mazes

Learning between the radiolarian and hexagon mazes was assessed by examining the interaction in a repeated-measures ANOVA comparing the first and last 3 days of trials. For total visits, there was no overall between-maze effect (*F*_(1,14)_ = 1.646, *p* = 0.220) but there was a significant interaction (*F*_(1,14)_ = 20.03, *p* = 0.001), reflecting a decrease in the total of number of visits in the hexagon maze but not in the radiolarian maze. For task latency, there was a significant between-maze effect (*F*_(1,14)_ = 1.409, *p* = 0.017) but no interaction (*F*_(1,14)_ = 2.72, *p* = 0.122).

Omission errors showed significant between-maze effects (*F*_(1,14)_ = 4.659, *p* = 0.049), in which mice exhibited both fewer omission errors and shorter trial times in the hexagon maze than the radiolarian maze, with no interaction (*F*_(1,14)_ = 2.02, *p* = 0.178; Fig. 4A).

Analysis of commission errors found a significant between-maze effect in which mice made fewer errors in the hexagon maze than the radiolarian maze overall (*F*_(1,14)_ = 7.621, *p* = 0.015). Additionally, the interaction with trial block was highly significant (*F*_(1,14)_ = 18.509, *p* = 0.001) due to a greater reduction in the hexagon maze than the radiolarian maze (Fig. 4B). We further split the commission error data into its component parts, re-entry and reference memory errors. For re-entry errors, there were significant between-maze effects overall (*F*_(1,14)_ = 6.525, *p* = 0.023) and a significant interaction (*F*_(1,14)_ = 16.738, *p* = 0.001), whereby re-entry errors decreased more on the hexagon maze (Fig. 4C). For reference memory errors, mice committed fewer errors in the hexagon maze than in the radiolarian maze overall (*F*_(1,14)_ = 36.036, *p* < 0.001), but there was no interaction (*F*_(1,14)_ = 1.724, *p* = 0.210; Fig. 4D).

Together, these data suggest that mice made fewer errors and learned more effectively on the hexagon maze than on the radiolarian maze.

#### 3.2.5 Summary

In summary, mice were able to learn a spatial reference memory task on both a 3D maze (the radiolarian maze) and a 2D maze (the hexagon maze). There were, however, differences in learning between mazes, with mice exhibiting more re-entry errors, and more reference memory errors on the three-dimensional maze than the two-dimensional maze. These results are further discussed below.

## 4 Discussion

Motivated by previous behavioural and electrophysiological findings, together with those from decades of behavioural research in two-dimensional environments using the radial arm maze, we developed a three-dimensional radial arm maze, the radiolarian maze, to test whether mice can represent locations distributed within 3D space and whether these representations can be maintained over time. Mice on the radiolarian maze learned both working and reference memory versions of the task, indicating that the animals could learn and remember, both within trials and across days, spatial locations distributed within 3D space. However, they did this less easily than a comparable task on a two-dimensional radial maze variant, the hexagon maze, which had been matched for spatial complexity. We conclude that mice can represent 3D space but with more difficulty than 2D space. Below, we examine the findings of the study and then discuss how the radiolarian maze may have been represented by the mice, together with possible reasons for the apparent difficulty in forming/using this representation.

### 4.1 Spatial performance in two versus three dimensions

Experiment 1 comprised a working memory task to see whether mice could represent the spatial layout of the mazes and hold this in short-term (working) memory. Because of the evident application of a procedural (motoric) rule in the classic maze, the results are not informative with regard to spatial encoding and so discussion is restricted to the other two mazes. Re-entry errors into already-visited arms declined significantly across the course of learning, indicating that mice were equally as able to hold a short-term representation of previously visited reward locations in the 3D radiolarian maze as they were on the 2D mazes. In Experiment 2, the reference memory task, mice also showed significant learning in both mazes and the rate of reduction of reference memory errors was the same. However, although the rate of learning was the same, the total number of both working memory and reference memory errors were greater in the radiolarian maze, with the reference memory errors—the best index of long-term spatial memory in these tasks—asymptoting at around 40% on the radiolarian maze. This suggests increased difficulty in forming, retaining or using a long-term representation of reward locations distributed through three-dimensional space.

That mice were able to learn the radiolarian maze suggests at least a partial ability to represent the vertical component of a spatial task, and so the question becomes how they do this, and why performance is less good than in two dimensions. There are several potential reasons for the lowered performance. One is simply that the physical demands of moving on a difficult surface, which required considerable effort not to fall off, and also to move around, might have diverted cognitive resources (including attention) away from the spatial components of the task and towards its more procedural aspects. The second is to do with limitations in how the mice cognitively represented the maze. The representational possibilities, and limitations, are discussed below, together with suggestions for future experiments to disentangle these hypotheses.

### 4.2 How the radiolarian maze might be represented

Informed by the findings of this study, together with those of recent behavioural and electrophysiological studies, we suggest three potential mechanisms by which the mice might have been able to represent the radiolarian maze task.

First, the mice might have created an integrated volumetric map for representing the 3D maze layout. In such a map, each goal location would have unique 3-component spatial coordinates (*x*, *y* and *z*), and/or each arm of the maze would be represented by a unique vector; animals could thus solve the task by visiting each of the relevant locations in the same way as they do on the standard radial maze. We initially considered it unlikely that they would do this: based on the neural findings of Hayman et al. [7] and of the behavioural findings of Grobéty and Schenk [5] and Jovalekic et al. [4] we thought it more likely that the animals would form an essentially planar representation of the maze layout, and thus (a) confuse arms having the same horizontal coordinates, and/or (b) solve the task in a planar way, visiting all the arms of one layer first followed by all the arms of the second layer, as has been seen in the previous behavioural studies. Our present findings suggest that arm confusion is unlikely given the equally fast learning of the working memory task in both 3D and 2D mazes. A planar representation might also seem unlikely because analysis of the pattern of responding on the radiolarian maze showed that arm choices were not distributed layer-wise; in fact, horizontal within-layer movements and vertical between-layer movements occurred equally often. However, if the mice had formed an integrated volumetric map then it seems surprising that they would have so much more difficulty with the reference memory task on the 3D maze than with its 2D counterpart, since the mazes had the same number of arms, and a truly integrated map should make no particular distinction between dimensions.

An alternative possibility is that the representation is planar, but it comprises two planes—one map for each layer—and the mice were forced, by the unusual affordances of the maze structure, to intersperse visits to one plane with those of the other. That is, sometimes the mouse would find itself positioned such that it was easier to swap layers than to work its way around an arm to the next arm in the same layer. If this is the case, then in the radiolarian maze, mice would have needed to access and integrate both of these maps at once, while in the hexagon maze mice were only required to access one horizontal map, with less cognitive load and hence faster learning. That they were unimpaired on the short-term (working) memory version of the task (Experiment 1) suggests that maybe the problem is with storing and retrieving the information across time. Alternatively, perhaps with the working memory task it is possible to create a more path-integration-based motoric representation of visited and not-yet-visited arms.

A possible intermediate between one volumetric versus two planar maps is a surface-coding one: that the mice form one large, planar map in which the surface of the spherical maze is “unwrapped” and represented in 2D coordinates. In this scenario, the position in absolute vertical space is disregarded, and the position of the arms is specified relative to the surface of the sphere and relies on metric information being applied to the curved plane of locomotion of the mouse. We cannot rule out this possibility using behavioural data alone; recordings of spatially sensitive neurons—especially grid cells [8] and head direction cells [9]—would be needed to test the surface-coding hypothesis. If such were the case, though, it seems surprising that there would be such a difference in learning between the radiolarian and hexagon mazes, unless the surface-coded map is somehow harder to consult due to the less orderly array of arms.

## 5 Conclusion and future work

For a full understanding of spatial cognition it is important to understand how animals represent and use space in all three of its dimensions; accordingly, the present experiment investigated whether mice could learn a 3D analogue of a common 2D laboratory spatial task, the radial maze. We found that they can learn this task but do so with greater apparent difficulty. Future work is needed to find out how they solve this task, and why learning is less effective than an apparently no-less-complex 2D maze. It may be that learning was affected by the increased locomotor challenges imposed by the 3D task, a possibility that could be addressed by creating a 2D analogue of the radiolarian maze in which the mice need to devote an equal amount of attention and effort to simply moving around; for example by introducing barriers or gaps or other impediments that force the animals to concentrate on how they move as well as where they are going. The representational explanations will require more sophisticated study design. In our view, the most likely representational explanation is that mice form two planar maps of the two layers of arms, and need to switch between these as they move between them with consequent interference, but an alternative possibility is that they use a volumetric map which—due to its increased capacity requirements—is simply harder to form and use. Experiments with 2D mazes of varying complexity and capacity requirements will be informative here, but the recording of grid and head direction cells will also be needed, in order to determine how the spatial map for the radiolarian maze is configured in 3D space. Either way, the finding that mice can solve a 3D radial maze task indicates that they possess the cognitive machinery to operate effectively in a complex 3D world.

## Competing interests

The lead author is a non-shareholding director of Axona Ltd.

## Authors’ contributions

JW and KJ conceived and designed the experiments; JW carried out most of the experimental work; EH, MF, BJ, MD, AK, AO and NZ contributed to experimental design and carried out some of experimental work; JW analysed the data; JW and KJ wrote the manuscript. All authors gave final approval for publication.

## Funding

The work was supported by BBSRC (BB/J009792/1), MRC (G1100669) and Wellcome (#083540) grants to KJ, and a BBSRC CASE studentship (BB/F015968/1) to JW in collaboration with Axona Ltd.

## Footnotes

1 Radiolaria are zooplankton having radial symmetry: see https://en.wikipedia.org/wiki/Radiolaria.
